# Modulating the tumor microenvironment in a mouse model of colon cancer using a combination of HIF-1α inhibitors and Toll-Like Receptor 7 agonists

**DOI:** 10.1007/s00210-024-03658-8

**Published:** 2024-11-30

**Authors:** Leila Rostamizadeh, Mina Ramezani, Hannaneh Monirinasab, Kobra Rostamizadeh, Mehdi Sabzichi, Seied Rafi Bahavarnia, Karim Osouli-Bostanabad, Fatemeh Ramezani, Ommoleila Molavi

**Affiliations:** 1 Department of Molecular Medicine, Faculty of Advanced Medical Science, Tabriz University of Medical Sciences, Golgasht Avenue, Tabriz, Iran; 2 Faculty of Medical Science, Department of Anatomical Science, Tarbiat Modares University, Tehran, Iran; 3 Core Research Laboratory, Tabriz University of Medical Sciences, Tabriz, Iran; 4 Department of Psychiatry and Behavioral Sciences, Department of Pharmacology, School of Medicine, University of Washington, Seattle, WA USA; 5 School of Medicine, Pharmacy, and Biomedical Sciences, University of Portsmouth, White Swan Road, Portsmouth, Portsmouth PO1 2DT UK; 6 Department of Pharmaceutical Biotechnology, Faculty of Pharmacy, Tabriz University of Medical Sciences, Tabriz, Iran; 7 Screening Laboratory, Blood Transfusion Organization, Tabriz, Iran

**Keywords:** Combination therapy, Colorectal cancer, HIF-1α, Immunotherapy, Tumor microenvironment, TLR, Chemotherapy, Gene therapy

## Abstract

The immunosuppressive tumor microenvironment (TME) plays a pivotal role in the response to various anticancer therapies, such as immune and chemotherapeutic agents. In this study, the synergistic effects of gene-targeting HIF-1α siRNA combined with Toll-Like Receptor 7 agonist on TME remodeling were investigated in a mouse model of colorectal cancer (CRC). A HIF-1α-specific siRNA duplex was formulated based on the ionic gelation of tripolyphosphate (TPP) with cationic chitosan (CH) as a nanoplex and evaluated in terms of size, charge, polydispersity index and gel retardation assay. MTT assay was conducted to assess the cytotoxicity of the specific siRNA duplex against CT26 cells. Hypoxic condition was generated to evaluate the gene and protein expression levels of HIF-1α, respectively. CT26 mouse model was established to assess the synergistic effect of silencing HIF-1α combined with oxaliplatin (OXA) and imiquimod (IMQ) on tumor growth. The mean diameter of the CH/siRNA nanoparticles was 243 ± 6 nm, as confirmed with Micrograph scanning electron microscope. There were no significant differences observed between the CT26 cells treated with nanoparticles alone and the untreated cells, indicating that these nanoparticles are safe and physiologically biocompatible (*p* ≥ 0.05). Triple combination therapy involving HIF-1α siRNA, OXA, and IMQ significantly retarded tumor growth and led to elevated levels of cytokines linked to cellular immunity (INF-γ and IL-12) compared with those in the other groups (*P* < 0.05). The positive correlation coefficient (r = 0.68) between tumor size and HIF-1α expression levels was statistically significant (*P* = 0.003). Compared with those in the control group, the expression levels of the anti-inflammatory cytokines IL-10 and IL-4 significantly decreased (*P* < 0.05). In conclusion, our findings suggest that inhibiting HIF-1α could serve as a rational strategy to enhance the antitumor response in the TME.

**Supplementary Information:**

The online version contains supplementary material available at 10.1007/s00210-024-03658-8.

## Introduction

Among multiple therapeutic combinations, chemoimmunotherapy has shown promising outcomes compared to alternative therapies. As immunotherapy stimulates and restores the immune system, chemotherapeutic agents provide tumor-associated antigens (TAAs) to trigger further antitumor immune responses (Waldman et al. 2020). Despite significant advances in cancer chemoimmunotherapy, the desired therapeutic benefits have not yet been fully realized (Le et al. 2019, Ning et al. 2021); therefore, synergistic strategies are still essential for most cancers.

The TME constitutes the internal milieu where tumor cells originate and reside. It encompasses not only the tumor cells themselves but also extracellular matrix elements, including fibroblasts, tumor-associated macrophages, T lymphocytes, adipocytes, mesenchymal stem cells, inflammatory cells, and other cell types. Immunosuppression within the TME has been pinpointed as a significant obstacle to the effectiveness of cancer immunotherapy (Pitt et al. 2016). Due to irregular angiogenesis, tumors are characterized by hypoxia, an acidic pH, and elevated pressure. Hypoxia, recognized as a key controller and catalyst for cancer progression and a significant feature of interactions between cancer cells and the TME, plays a dynamic role in both metastasis and resistance to treatment (Mo et al. 2021). In terms of molecular mechanisms, the proliferation and survival of cancer cells within a hypoxic TME are mainly supported by the hypoxia-inducible factor (HIF) family of transcription factors. (Petrova et al. 2018, Wang et al. 2021). Several studies have demonstrated that high levels of *HIF-1α* are associated with tumor metastasis, angiogenesis, poor prognosis, and resistance to therapy (Balamurugan 2016, Jögi et al. 2019). Therefore, targeting *HIF-1α* as part of combination therapy may lead to augmented antitumor effects and improved outcomes (Kessler et al. 2010, Liao et al. 2012). Toll-like receptor (TLR) agonists induce robust innate and adaptive immune responses against tumor cells (Chen et al. 2022). The function of immune cells within the TME is often immature due to the suppressive impact of *HIF-1α* overexpression. However, triggering dendritic cells (DCs) using Toll-like receptor (TLR) agonists leads to increased expression of costimulatory molecules and inflammatory cytokines. This process has demonstrated efficacy in reprogramming and remodeling the TME (Augustin et al. 2020). Imiquimod (IMQ), a TLR7 agonist, has both direct and indirect anticancer effects through stimulating tumor cell apoptosis and inducing potent immune responses (Huang et al. 2020). The synergistic effects of HIF inhibitors combined with immunoadjuvants have been demonstrated in several studies. Preclinical studies have shown that intratumoral (i.t.) application of IMQ modulates the TME by increasing the number of effector immune cells, cytotoxic T lymphocytes (CTLs), and mature dendritic cells (mDCs) and decreasing the number of suppressor immune cells (MDSCs; myeloid-derived suppressor cells and Tregs; T regulatory cells) (Pilch et al. 2018). Huang et al. showed that genetic silencing of *HIF-1α* sensitizes tumor cells to IMQ treatment. Based on these findings, *HIF-1α* inhibition may be a potent therapeutic option to enhance the antitumor effects of cancer immunotherapy (Huang et al. 2014).

Recently, nanotechnology has undergone notable advancements, providing multiple benefits in combination therapy (Patra et al. 2018, Mohammadian et al. 2020, Chavoshi et al. 2023, Alhazami et al. 2024). In this context, chitosan (CH), a natural polymer, is highly appealing as a vector for nucleic acid delivery in biological applications due to its biocompatibility (Cao et al. 2019). Several preclinical studies have provided evidence that CH nanoparticles are a safe and effective strategy for siRNA delivery to tumor cells in vivo (Mainini and Eccles 2020). Harnessing the disruption of key RNAs via RNA interference (RNAi) has emerged as a promising therapeutic modality for cancer treatment (Li et al. 2023). Therefore, we used CH/*HIF-1α* siRNA nanoplexes to *inhibit HIF-1α *in vitro and in vivo.

Here, we propose that *HIF-1α* inhibition via siRNA technology can overcome the immunosuppressive TME and enhance the therapeutic efficacy of chemotherapy (OXA) and immunotherapy (IMQ) in murine mouse models of CRC. This regime guarantees the establishment of a favorable TME with adequate levels of TAA and TLR agonists for immune cells, promoting immune cell activation, overcoming tumor tolerance, and fostering robust antitumor immunity.

## Materials and methods

### Materials

Low-molecular-weight chitosan (CH; 114 kDa, 84% deacetylation; Sigma‒Aldrich) was used for nanoparticle preparation. A 22-mer *HIF-1α*-specific siRNA duplex containing the following sequences was obtained from BIONEER Corporation (Korea): murine *HIF-1α* sense, 5′-CAGUUACGAUUGUGAAGUU-3′, and antisense, 3′-AACUUCACAAUCGUAACUG-5′. IMQ powder was purchased from InvivoGen (San Diego, USA). Western blotting antibodies were procured from Santa Cruz Biotechnology (*β-Actin* (C4): sc-47778, *HIF-1α* (28b): sc-13515, *Bax* (B-9): sc-7480, *IFN-γ* (A-9): sc-390800) and BioLegend (San Diego, USA) (anti-*STAT3* Phospho (Tyr705): Cat-651001). BALB/c mice and the murine CRC cell line CT26 were obtained from Pasteur Institute (Tehran, Iran).

### Preparation of CH nanoparticles

CH nanoparticles were generated via the ionic gelation of tripolyphosphate (TPP) with cationic CH. To prepare the CH working solution (2.1 mg/mL), 10.5 mg was dissolved in 5 mL of acetic acid solution (0.25%, pH 4.5) while stirring. To promote further dissolution, the solution was subjected to dry ultrasonication for 3 min. NPs were formed by gradually introducing TPP (0.3%) to the CH solution while stirring at room temperature. The characteristics of the nanoparticles (size, surface charge (zeta potential), and polydispersity index (PDI)) were determined by dynamic light scattering (DLS) using a Malvern Zeta-sizer Nano-ZS ZEN. To assess the structure, morphology, size, and purity of nanoparticles, a scanning electron microscope (MIRA3 FEG-SEM, Vega Tescan, Czech Republic) was done. The samples were diluted with 10 μL of distilled water, air-dried under vacuum, mounted on an aluminum stub, coated with gold using a sputter coater.

### Preparation of CH/siRNA nanoplexes

The CH/siRNA nanoplexes comprise a positively charged CH nanoparticle and a negatively charged siRNA. Compared to other nanostructures, these nanoplexes demonstrate superior yield and complexation efficiency (Serrano-Sevilla et al. 2019). The specific N/P ratios, representing the molar ratio of CH amino groups to RNA phosphate groups, were calculated using the following formula to achieve optimal siRNA loading on the nanoparticles (Barichello et al. 2010). To prepare CH/siRNA nanoplexes with maximum encapsulation, the desired volume of CH solution was added to the siRNA solution at various N/P ratios ranging from 0 to 60. The mixture was then incubated at room temperature for 30 min. The resulting CH/siRNA nanoplexes were then utilized for subsequent experiments.

$$CH(\mu g)=\frac{(\mu g\;of\;siRNA\times nmol\;of\;phosphate\;per\;\mu g\times desired\;N/P\;ratio)}{nmol\;of\;CH\;per\;\mu l}$$

### CH/siRNA nanoplex gel retardation assay

The loading ability of siRNA into the nanoparticles was evaluated using gel retardation analysis. For this purpose, solutions of CH/siRNA nanoplexes were prepared at different N/P ratios. Subsequently, the free siRNA and CH/siRNA solutions were loaded onto a 2% agarose gel and electrophoresed in 1 × TBE buffer (pH 7.2) at 80 V for 45 min. Finally, the CH/siRNA nanoplexes were visualized on a gel documentation system (Qiagen) following staining with a safe stain.

### Cytotoxicity study of the CH/siRNA nanoplexes

The cytotoxicity of the nanoparticles was studied using the MTT assay (Tupal et al. 2020). To do this, CT26 cells were seeded at a density of 1 × 104 cells/well in a 96-well plate with a total volume of 100 µL of RPMI medium (Gibco®) containing 10% FBS and 1% penicillin/streptomycin and placed in an incubator at 37 °C with 5% CO2 for 24 h. The cells were then treated with serial dilutions of the nanoparticles. After incubation for 48 h, MTT reagent (5 mg/mL, Sigma) was added to each well at a 1:3 dilution and incubated for 4 h. The medium was then replaced with DMSO (Sigma), and the relative absorbance (at 570 nm) was measured using an ELISA plate reader (BIOTEK ELX800TS).

### Synergistic effects of triple combination treatment with OXA, IMQ, and HsiRNA on CT26 cells

Chou and Talalay analysis were performed to determine the effects (additive, synergistic, or antagonistic) of combination treatment on tumor cells (Chou 2010). CT26 colon cancer cells were sequentially exposed to drugs alone and various combinations of OXA, IMQ and HIF-1α siRNA at a nonconstant ratio. Compared with drug-alone treatment, combination therapy exhibited more than an additive effect.

### Hypoxic induction of CT26 cells with CoCl_2_

CoCl2 (cobalt (II) chloride) was used to induce chemical hypoxic TME in the cell culture. Hypoxic conditions were simulated by incubating the cells with 25, 50, 100, 150 μM CoCl2 for 24 h to determine the optimal dose (Rana et al. 2019). Subsequently, the expression levels of *HIF-1α* were evaluated by real-time PCR (qPCR).

### HIF-1α gene silencing in CT26 cells

The silencing activity of the CH/*HIF-1α* siRNA nanoplexes in the CT26 tumor cell line was investigated using qPCR. To this end, 1.5 × 10^5^ CT26 cells were seeded into a 6-well culture plate containing RPMI medium (10% FBS and 1% penicillin/streptomycin) and incubated at 37 °C in a 5% CO2 environment for 24 h. Subsequently, to induce a model hypoxic response, 24 h before CH/*HIF-1α* siRNA transfection, the medium was replaced with 2 ml of fresh medium containing 100 µM CoCl_2_ and 5% FBS without antibiotics. Transfection was performed on cells that were approximately 70% confluent. On the day of transfection, the cells were treated with CH/*HIF-1α* siRNA nanoplexes (N/P = 60) at various *HIF-1α* siRNA concentrations (25, 50, 75, and 100 nM) in 500 µL of FBS-free media. After 4 h, 500 µL of fresh medium containing 20% FBS and 100 µM CoCl_2_ was added to the transfected cells in each well and incubated for 48 h. The expression levels of *HIF-1α* mRNA were subsequently assessed by qPCR, while transfection with PBS or *HIF-1α* siRNA alone served as a control.

### Establishment of a CT26 syngeneic mouse model of CRC

Female BALB/c mice, aged 5–6 weeks, were maintained in a pathogen-free environment. Syngeneic tumor-bearing mice were generated by subcutaneous (s.c.) implantation of 5 × 10^5^ CT26 cells in 100 μL of PBS into the right flank of the mice (Takahito Taniura et al. 2020). On days 12–16, when the tumors became palpable, the mice were randomly divided into six treatment groups (*n* = 4): 1) PBS (control), 2) CH nanoparticles, 3) OXA + IMQ, 4) *HIF-1α* siRNA + OXA, 5) *HIF-1α* siRNA + IMQ, and 6) *HIF-1α* siRNA + IMQ + OXA (triple combination). OXA was administered intraperitoneally (i.p.) at a dose of 5 mg/kg, and IMQ and siRNA-*HIF-1α* were injected i.t. at doses of 25 µg/mouse and 50 pmol/mouse, respectively, on alternate days. CH nanoparticles and PBS were injected i.t. at 0.1 ml/mouse daily. This procedure was repeated for 10 days, during which the tumor size was measured using a caliper every two days. The tumor volume was calculated using the following formula: tumor volume (mm^3^) = d^2^ × D/2, where d and D are the short and long diameters of the tumors, respectively. After the 10-day intervention, all animals were euthanized, and tumor samples were removed, photographed, and stored at −70 °C for further analysis.

### Quantitative real-time PCR

Total RNA was extracted using the chloroform-isopropanol isolation method. A NanoDrop 2000C spectrophotometer (USA) was used to assess the quality and quantity of the isolated RNA (Ahmadian et al. 2021). Subsequently, qPCR was performed using SYBR Green I master mix on a qPCR thermal cycler (Roche, Germany) to evaluate the relative expression levels of the target genes. β-actin was used as a normalizing gene to calculate the corresponding cycle threshold (Ct). The primers used in this study are listed in Table 1.

**Table 1:** Primers used for real time qPCR

Gene	Primer sequence
BAX	F: 5’-AGGGTTTCATCCAGGATCGAG -3’
	R: 5’-TCCACGTCAGCAATCATCCTC -3’
BAD	F: 5’-GCAGCCCAGAGTATGTTCCAG -3’
	R: 5’-CGTCCCTGCTGATGAATGTTG -3’
Bcl-2	F: 5’- GGAGGATTGTGGCCTTCTTC -3’
	R: 5’-GCCCAATACGACCAAATCCGTTG-3’
HIF-1α	F: 5’-TTTGGACACTGGTGGCTCAG -3’
	R: 5’-GAGCTGTGAATGTGCTGTGATC -3’
IL-10	F: 5’- CCTGGGTGAGAAGCTGAAGAC-3’
	R: 5’-ATGGCCTTGTAGACACCTTGG -3’
IL-12	F: 5’-CTATGGTCAGCGTTCCAACAG -3’
	R: 5’-AGGTGGTTTAGGAGGGCAAG -3’
INF-* γ*	F: 5’- ACAATGAACGCTACACACTGC-3’
	R: 5’-CATCCTTTTGCCAGTTCCTCC -3’
STAT3	F: 5’-AAACCCTCAAGAGCCAAGGAG -3’
	R: 5’-ACGTACTCCATTGCTGACAAG -3’
VEGF	F: 5’-CTGCCTGGAAGAATCGGGAG -3’
	R: 5’- GTACCCAGGAGGTGGGGTAA-3’
IL-4	F: 5’- CGAGTTGACCGTAACAGACAT-3’
	R: 5’- CGTCTTTAGCCTTTCCAAGAAG -3’
β-actin	F: 5’- ACCCACTCCTCCACCTTTG-3’
	R: 5’- CTCTTGTGCTCTTGCTGGG -3’

### Western blot analysis

After treatment and tumor excision, the samples were lysed on ice. The extraction buffer was centrifuged at 12,000 rpm for 10 min at 4 °C and then subjected to sodium dodecyl sulfate‒polyacrylamide gel electrophoresis (SDS‒PAGE) using a 10% polyacrylamide gel. Immunoblotting was performed with specific antibodies to assess the relative expression of the desired proteins. The relative intensity of the relevant bands was normalized to the *β-actin* intensity in the same sample using ImageJ (Schneider et al. 2012).

### Statistical analysis

All the data are expressed as the mean ± standard deviation (SD). For comparison of means, analysis of variance (ANOVA) was used. All analyses were conducted using GraphPad Prism version 9 software. Significance is shown as follows: *P < 0.05, **P < 0.01, ***P < 0.001, ****P < 0.0001.

## Results

### Preparation and characterization of the nanoparticles

**Fig. 1:**
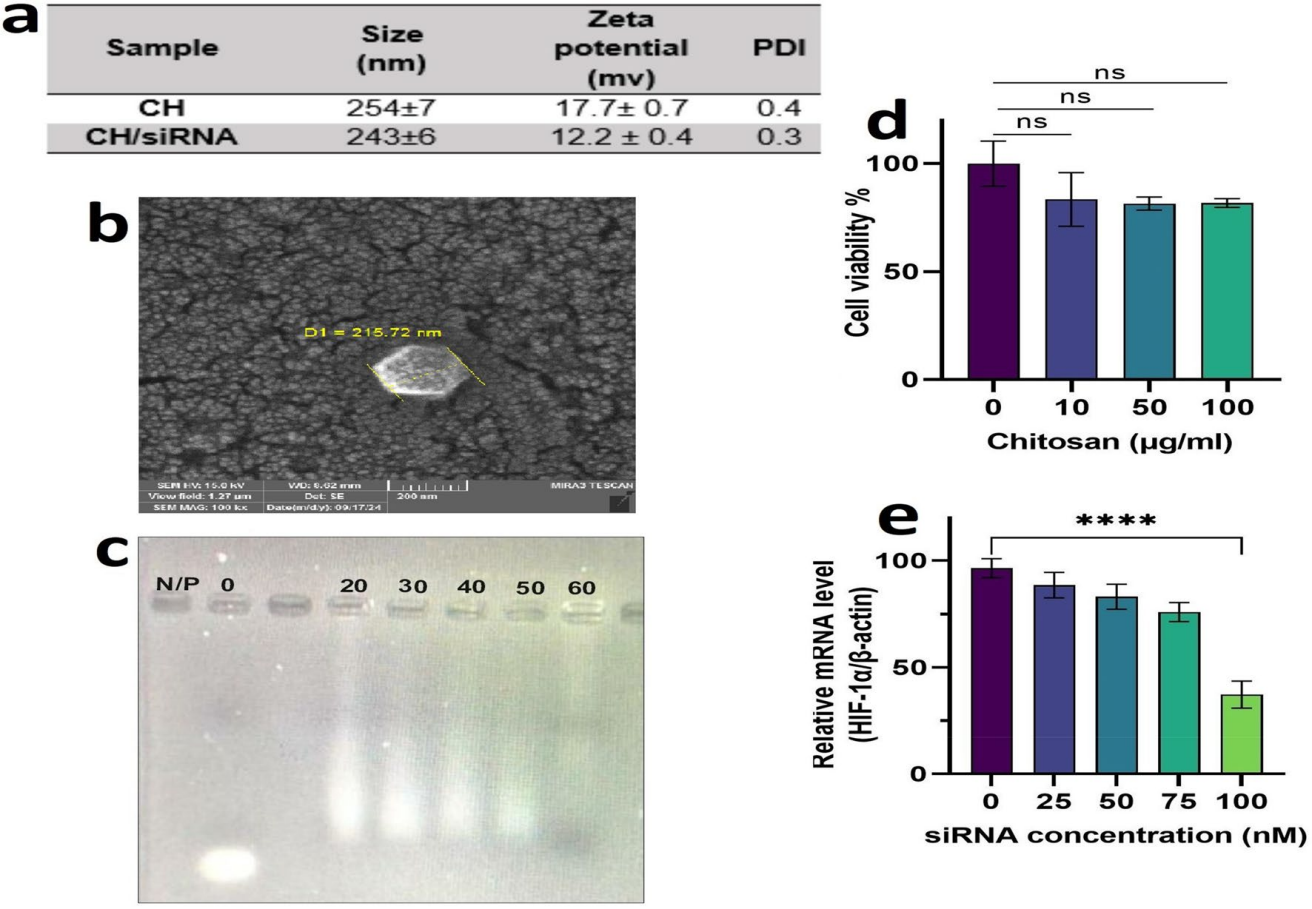
Characterization of CH/HIF-1α siRNA nanoparticles: **a** Characterization of the size, zeta potential, and PDI of both CH nanoparticles and CH/HIF-1α siRNA nanoplexes. **b** Scanning electron microscopic (SEM) graph of CH/HIF-1α siRNA nanoplexes. **c** The gel retardation assay is presented here, evaluating CH/siRNA nanoparticles with varying N/P ratios. **d** The viability of CT26 cells was assessed following incubation with different concentrations of CH nanoparticles alone and CH-siRNA. **e** The efficiency of HIF-1α siRNA silencing by CH nanoplexes in CT26 cells was explored. Tumor cells were cultured and transfected with CH/HIF-1α siRNA nanoplexes containing different concentrations of HIF-1α siRNA (25, 50, 75, and 100 nM) at an N/P ratio of 60. After 48 h of incubation, HIF-1α mRNA levels were measured using qPCR. Beta-actin was used as a housekeeping gene for normalization. All the data are presented as the means ± SDs of three independent measurements

The CH/HIF-1α siRNA nanoplexes were formed by incubating the nanoparticles with the siRNA molecules. Figure 1a and b shows the physical characteristics of both the isolated nanoparticles and the resulting CH/HIF-1α siRNA nanocomplexes. The CH/siRNA nanoparticles exhibited an average diameter of 243 ± 6 nm, indicating a relatively acceptable size distribution (PDI = 0.3 ± 0.04) which with uniform shape in the range of 200–250 nm approved by SEM. Furthermore, the decrease in zeta potential to 12.2 ± 0.4 mV after siRNA incorporation confirmed the successful formation of the CH/siRNA nanoplexes. To determine the proper N/P ratio to achieve maximum encapsulation, a gel retardation assay with CH/siRNA nanoplexes at different N/P ratios (20–60) was performed. The results indicated that complete complexation of siRNA was achieved at an N/P ratio of 60 (Fig. 1c).

### Cytotoxicity of the nanoparticles in CT26 cells

Figure 1d displays the cell viability after incubation with various concentrations of nanoparticles, ranging from 10 to 100 μg/ml. Compared with that of untreated cells, the viability of cells incubated with nanoparticles was 85%, indicating good biocompatibility and low cytotoxicity.

### Synergistic effects of triple combination treatment with OXA, IMQ, and HsiRNA on CT26 cells

As shown in Table 1, in all the different combination treatments, the CI values were less than 1, indicating synergistic effects between the drugs at low doses. At some concentrations, combination therapy resulted in a CI of almost 0.6, which in turn suggested a strong synergistic effect.

**Fig. 2:**
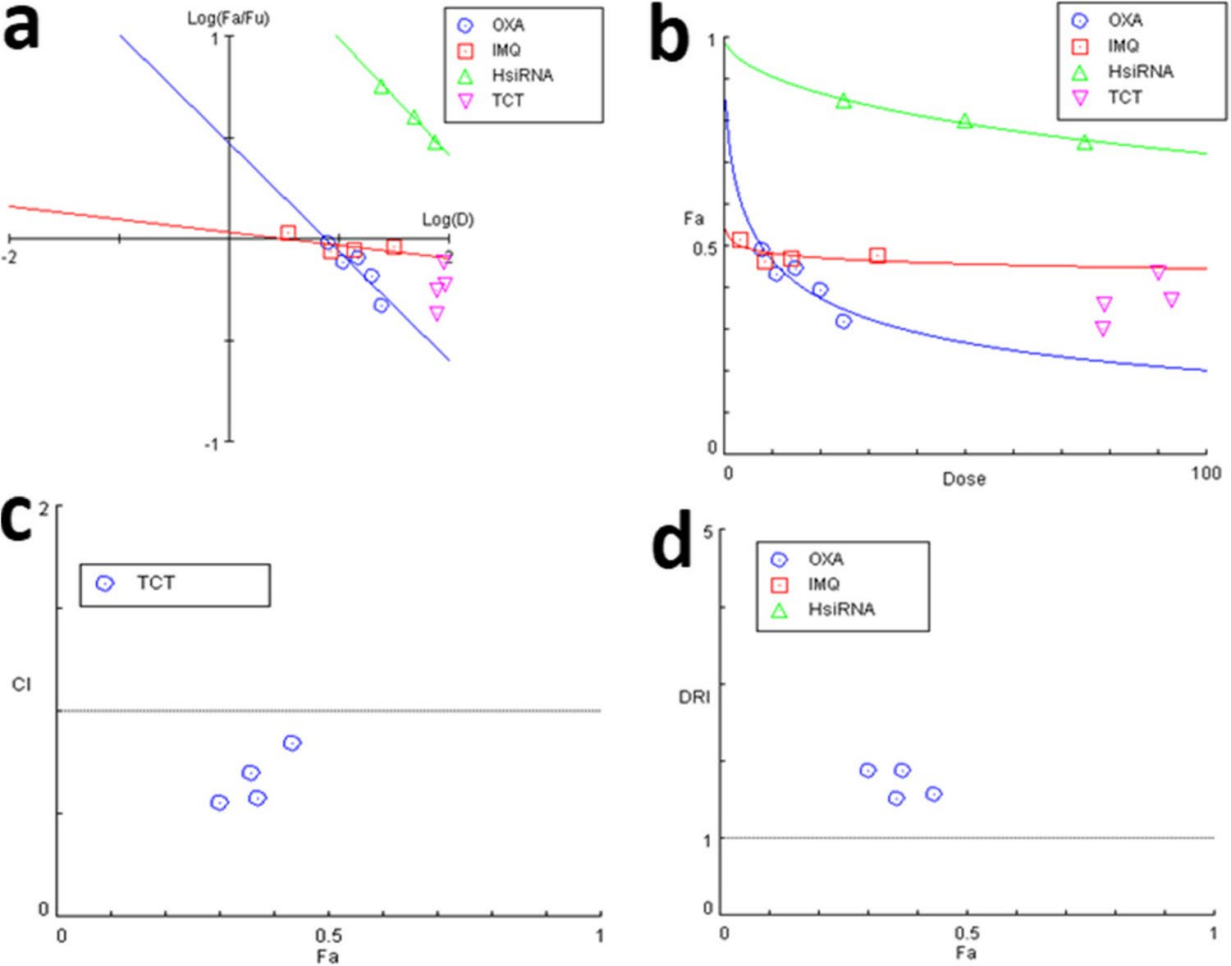
Combination analysis of OXA, IMQ, and HsiRNA combination therapy on CT26 colon cancer cells. **a** Dose‒effect curve, **b** median-effect plot for single and triple combination therapy (TCT), **c** combination index plot, and **d** DRI plot for four combinations of OXA, IMQ & HsiRNA on CT26 colon cancer cells

A median effect plot was generated to determine the pharmacological median doses for lethality (LD50), toxicity (TD50), and the effect of agonist drugs (ED50) (Fig. 2a). To demonstrate the effects at different Fa values, CI values were calculated for each Fa and plotted in the FA-CI plot, as shown in Fig. 2b. As shown in Fig. 2c, the CI in all the different combinations was less than 1. The drug reduction index (DRI) is a measure of how much concentration of each drug in a synergistic combination may be reduced at a given effect level compared to single drug doses. A DRI = 1 represents no dose reduction, whereas DRIs > 1 and < 1 indicate favorable and negative dose reductions, respectively. In our study, at Fa = 0.435, the DRIs were 1.57, 6.55, and 16.89 for OXA, IMQ, and HIF-1α siRNA, respectively, indicating that combination therapy of CT26 cells could reduce the OXA dose up to 1.57 times while decreasing the IMQ dose up to 6.55 and the HIF-1α siRNA dose up to 16.89 times, respectively. Similarly, at other Fa values, a combination regimen could reduce the concentration of all drugs needed to kill tumor cells (Fig. 2d and Table 2).

**Table 2:** Data of combination therapy from OXA, IMQ & HIF-1 siRNA nM

No	OXA µM	IMQ µM	HIF-1 siRNA nM	CI value
1	8 (10)	32 (90)	50	0.84
2	11 (20)	32 (90)	50	0.57
3	15 (30)	14 (80)	50	0.69
4	20 (40)	8.5(10)	50	0.55

The value of CI (combination index), of C26 cell lines treated with four different concentrations of Oxaliplatin (OXA), Imiquimod (IMQ) and HIF-1 siRNA after 24 h incubation

CI < 1: synergistic effect, CI = 1 additive effect, CI > 1: antagonistic effect

### In vitro HIF-1α gene silencing by CH/HIF-1α siRNA nanoplexes

Relative *HIF-1α* mRNA levels were evaluated in CT26 cells incubated with CH/*HIF-1α* siRNA nanoplexes of different molarities (25, 50, 75, and 100 nM) at an N/P ratio of 60. After 48 h, gene expression was analyzed by qPCR. As illustrated in Fig. 1e, *HIF-1α* mRNA levels were significantly lower in the 100 nM siRNA group than in the other concentration groups and the control group (*P* < 0.0001).

### Animal model study

#### Synergistic effects of combination therapy on tumor growth inhibition

**Fig. 3:**
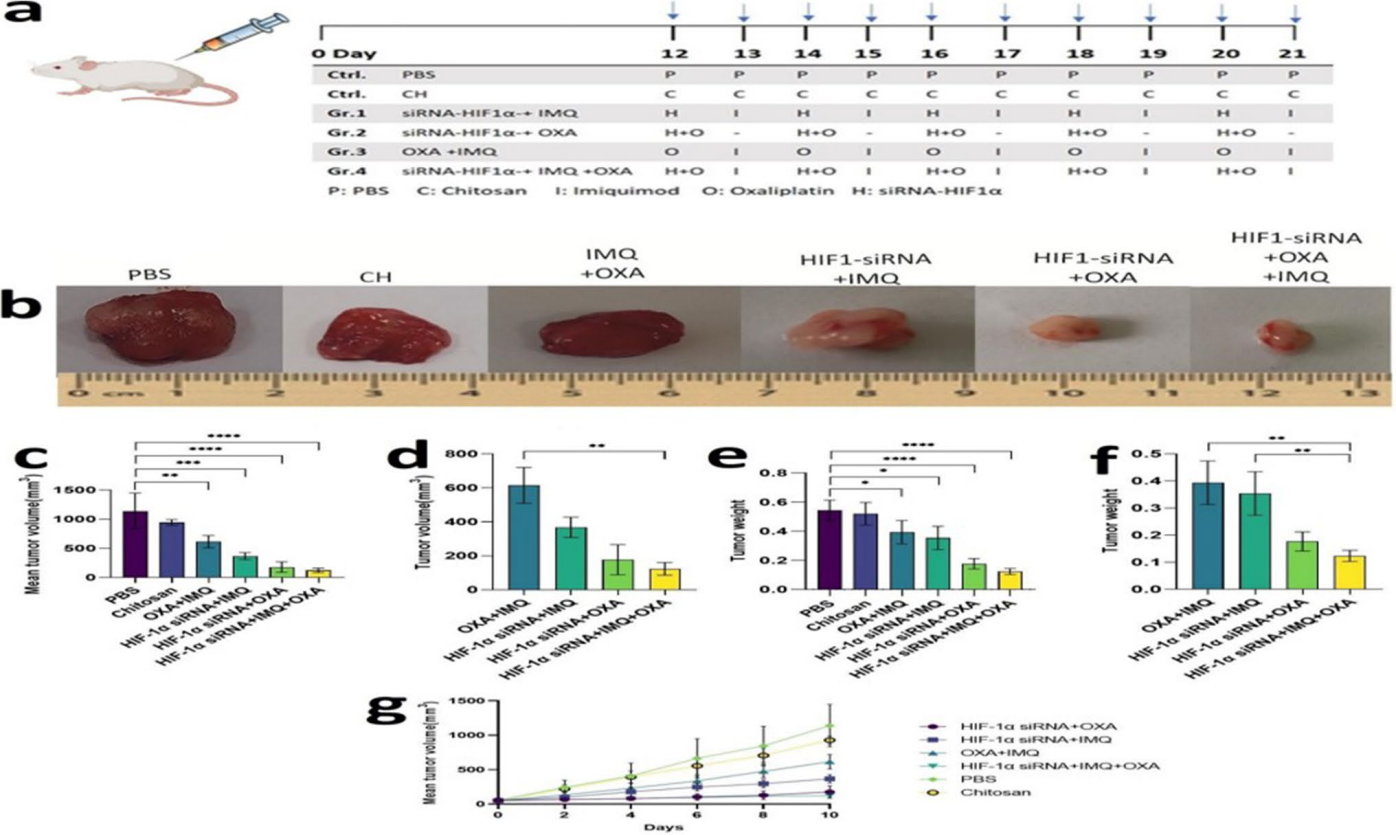
Effects of Combination Therapy on Tumor Weight and Volume in a Mouse Model of CRC. **a** Treatment protocol depicting the schedule for combination therapy designed to treat CT26 tumors. Treatments began 12–16 days after tumor implantation and were administered every two days. **b** Images of tumors harvested from untreated and treated mice. Resected tumors demonstrated that triple combination therapy resulted in a greater reduction in tumor size than dual therapy. **c** and **e**: Effects on tumor volume and weight. All combination therapies significantly reduced tumor volume and weight compared to those in the control group. **d** and **f** Double vs. triple therapy. These panels compare the effects of double and triple therapy. The largest reductions in both tumor volume and weight were observed in mice treated with the triple combination. **g** Tumor volume over time, indicating that triple combination therapy was more effective than dual therapy in suppressing tumor growth. Each line represents a specific treatment group and was plotted to show the primary tumor size relative to the days after tumor challenge

The therapeutic efficacy of the combination therapy was evaluated in BALB/c mice bearing subcutaneous CT26 tumors receiving OXA, IMQ, and HIF-1α siRNA according to the treatment regimen outlined in Fig. 3a. Notably, all mice tolerated the administered doses and treatment schedule of OXA, IMQ, and HIF-1α siRNA without adverse effects.

Before studying how combination therapy affects tumor growth, the impact of combination therapy on tumor phenotypes was examined. As shown in Fig. 3b, all the combination treatments reduced the tumor volume. While tumors in untreated mice grew significantly, those treated with dual or triple combinations together showed a substantial decrease in tumor growth. Tumor volume was significantly decreased in all treatment groups compared with that in the PBS or CH groups (Fig. 3c , *P* < 0.0001). Among the treatment groups, the smallest tumor size was observed in mice treated with the triple combination, whereas the largest size was observed in the OXA + IMQ combination group. Compared to that in the control group, the tumor volume in the treated group decreased 3.4-fold. In contrast, triple combination therapy reduced the tumor volume by 3.7 times compared to dual treatment.

The triple combination significantly diminished tumor size in CRC mouse models but did not result in complete tumor regression. The comparison between triple and double combination treatments revealed that the tumor size in mice treated with triple combination therapy was significantly lower than that in mice treated with double combination therapy, and this difference was statistically significant compared with that in the IMQ + OXA group (Fig. 3d , *p*< 0.01). Overall, triple therapy reduced tumor volume more than dual therapy.

Combination therapy significantly diminished the average tumor weight in all treatment groups. The average tumor weights decreased in mice exposed to IMQ combined with *HIF-1α* siRNA or OXA compared to those in the PBS and CH groups (Fig. 3e , *P*< 0.05). Notably, compared with the control treatment, the combination of *HIF-1α* siRNA + OXA and triple combination therapy resulted in a more marked reduction in tumor weight. Overall, the tumor weights in the combination treatment groups were two times (48%) lower than those in the control group, with the lowest weight in the triple therapy group. In particular, the tumor weight in the triple therapy group was three times (75%) lower than that in the dual therapy group, as shown in Fig. 3f (*P* < 0.01). The combination therapy gradually decreased tumor growth from day 6 to the final observation day (day 10), and the mean tumor volume was significantly reduced (Fig. 3g).

#### The effects of CH/HIF-1α siRNA nanoplexes on HIF-1α downregulation in a CT26 syngeneic mouse model

**Fig. 4:**
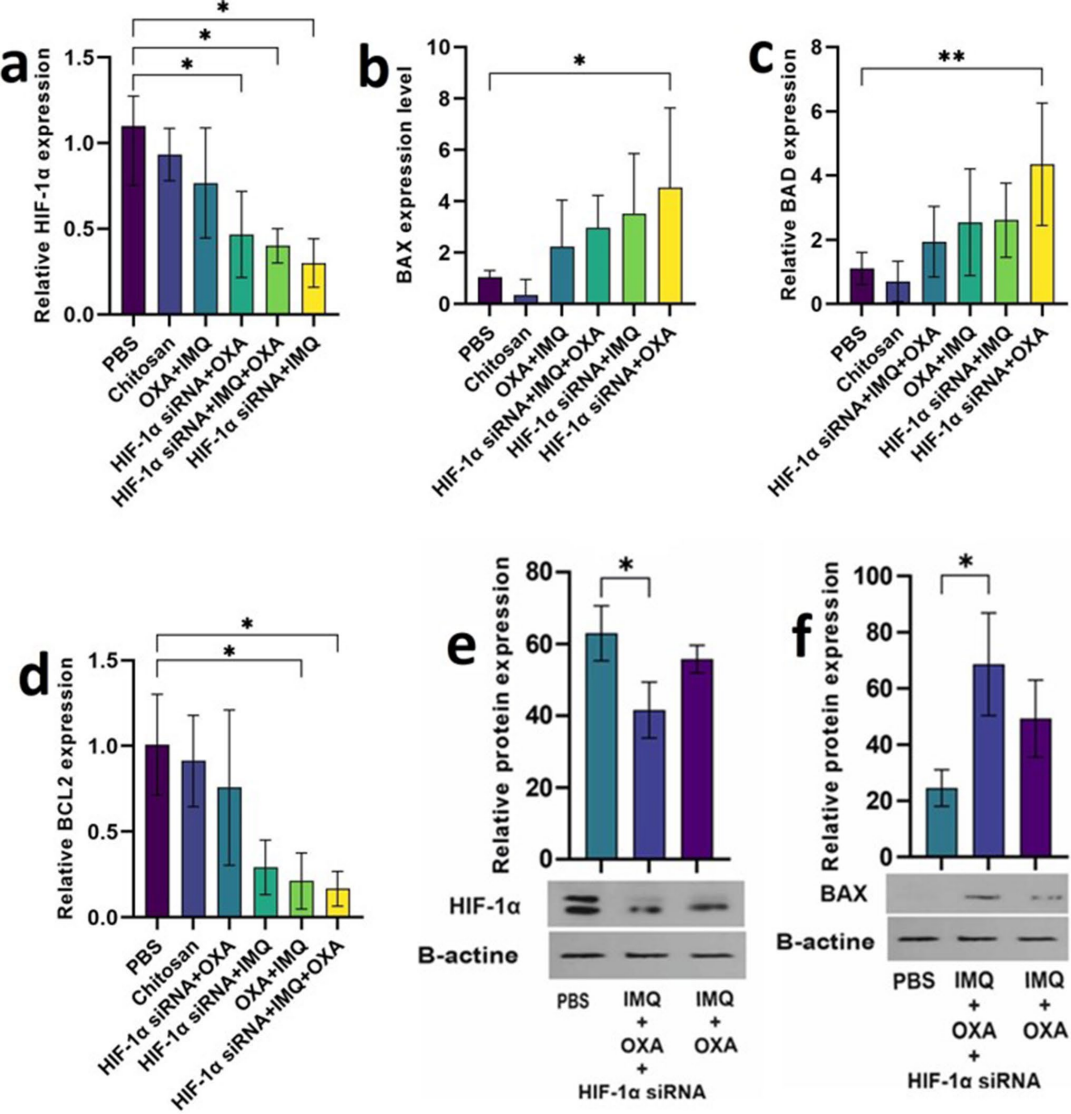
Changes in HIF-1α and Apoptosis-Related Gene Expression during Combination Therapy. **a** to **d** The impact of CH/HIF-1α siRNA nanoplexes on HIF-1α, BAX, BAD and BCL2 expression in a mouse model of colorectal cancer (CRC). **e** & **f** Investigation of how combination therapy affects the mRNA and protein levels of HIF-1α and BAX. All the data are presented as the mean ± standard deviation (SD) of the gene expression levels

The inhibitory effects of i.t. injection of CH/HIF-1α siRNA nanoplexes on the mRNA and protein expression levels of HIF-1α in tumors were investigated. As shown in Fig. 4a, the relative *HIF-1α* mRNA levels were markedly lower in the tumor tissue from mice injected with the CH/*HIF-1α* siRNA nanoplexes than in that from the uninjected group (*P* < 0.01). Among the treatment groups, the OXA + IMQ group had the highest *HIF-1α* expression, whereas the triple combination group had the lowest HIF-1α expression (Fig. 4b , *P*< 0.05). Furthermore, western blot analysis revealed a significant decrease in *HIF-1α* protein levels in *HIF-1α* siRNA-treated tumor tissues compared to those in untreated tissues (Fig. 4c , *P*< 0.05).

#### Effect of combination therapy on apoptosis stimulation

The impact of combination therapy on tumor cells was further investigated by analyzing the mRNA and protein expression levels of genes associated with apoptosis in CT26 tumor samples. The mRNA expression levels of proapoptotic genes, such as *BAD* and *BAX,* were significantly higher in the *HIF-1α* siRNA + OXA group than in the control group (Fig. 4d , *P*< 0.01). While the *BAX* expression levels were not significantly different between the triple*-* and double-injection therapy groups, the *BAD* expression levels were significantly higher in the *HIF-1α* siRNA + OXA group than in the triple-injection therapy group (Fig. 4e, g, and h; *P* < 0.05). Protein expression analysis revealed that the *BAX* levels in the mice treated with the triple combination agent were greater than those in the mice treated with the double combination agent (IMQ + OXA), which was significantly different from that in the control group (Fig. 4f , *P*< 0.05).

The mRNA expression levels of *BCL*−2, an antiapoptotic gene, were significantly lower in tumors from the triple combination and OXA + IMQ groups than in those from the untreated group (data not shown). Additionally, a comparison between triple and double combination therapy revealed that the expression of *BCL-2* significantly decreased in the triple therapy group compared to that in the *HIF-1α* siRNA + OXA group (results not shown).

#### Effect of combination therapy on cell proliferation

**Fig. 5:**
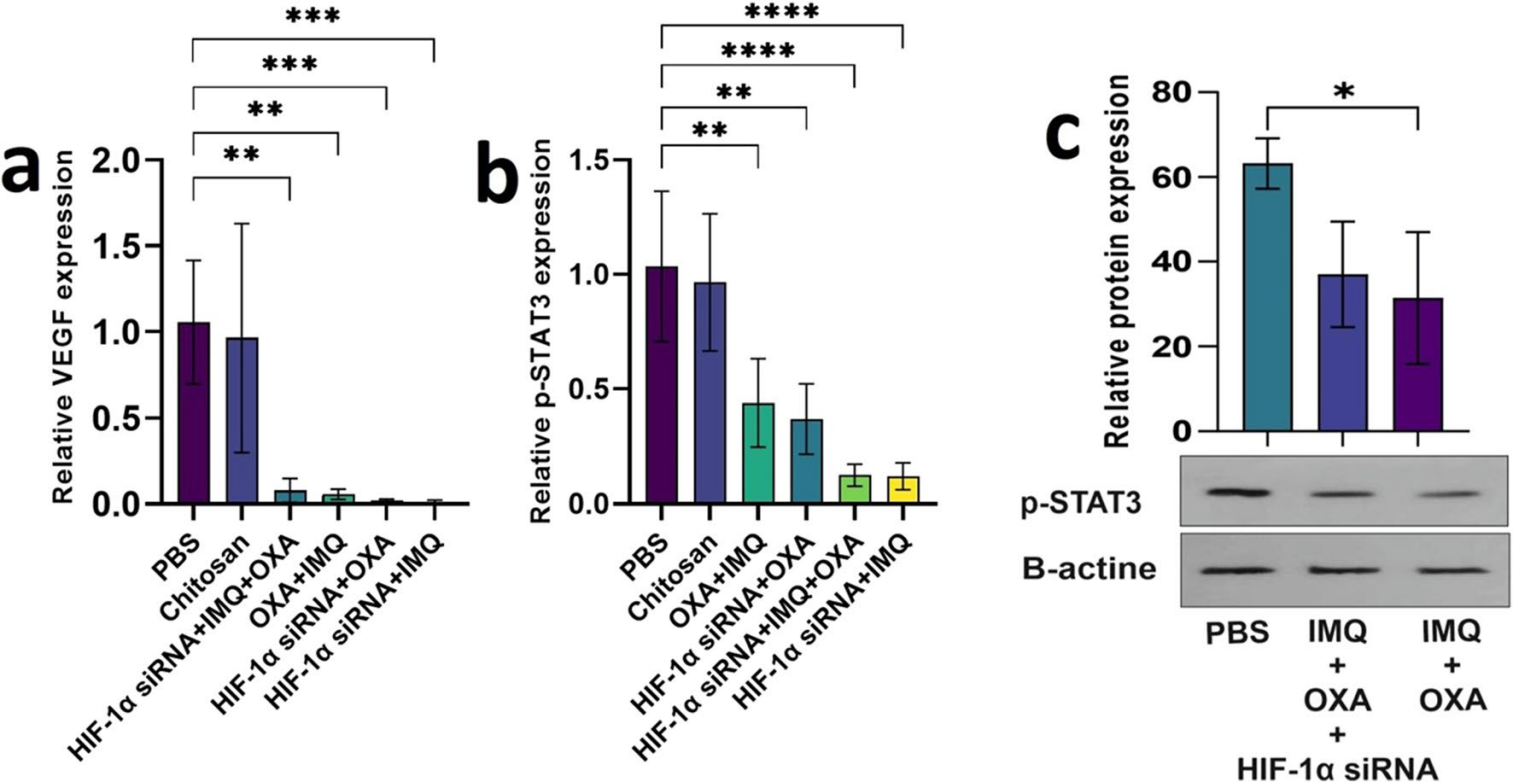
This figure explores how combination therapy affects the expression of genes involved in cell proliferation. **a** and **b** depict the mRNA expression levels of vascular endothelial growth factor (VEGF). **d** and **e** show the mRNA expression levels of phosphorylated signal transducer and activator of p-STAT3. **c** The protein expression levels of p-STAT3. Beta-actin, a housekeeping gene, was used for data normalization. All the data are presented as the mean ± standard deviation (SD) of the gene expression levels

For the initial assessment, we investigated the mRNA expression profiles of the phosphorylated forms of signal transducer and activator of transcription 3 (p-*STAT3*) and vascular endothelial growth factor (*VEGF*) in tumor tissue specimens after treatment. The results indicated that the mRNA expression levels of p-*STAT3* and *VEGF* were significantly lower in all the mouse-treated groups than in the PBS and nanoparticle groups (Fig. 5a and d , *P*< 0.0001).

Although *VEGF* expression levels were not significantly different between the triple and double therapy groups, p-*STAT3* expression levels were significantly lower in the triple therapy group than in the *HIF-1α* siRNA + OXA and OXA + IMQ groups (Fig. 5b and e , *P*< 0.05). Furthermore, analysis of the protein expression levels revealed that the p-STAT3 protein level in the mice treated with OXA + IMQ decreased significantly (Fig. 5c , *P*< 0.05). Our findings demonstrated that the combined therapy effectively downregulated proliferative genes, leading to the inhibition of tumor growth.

#### Combination therapy induces immune cell-related cytokine and Th1 polarization in the TME

**Fig. 6:**
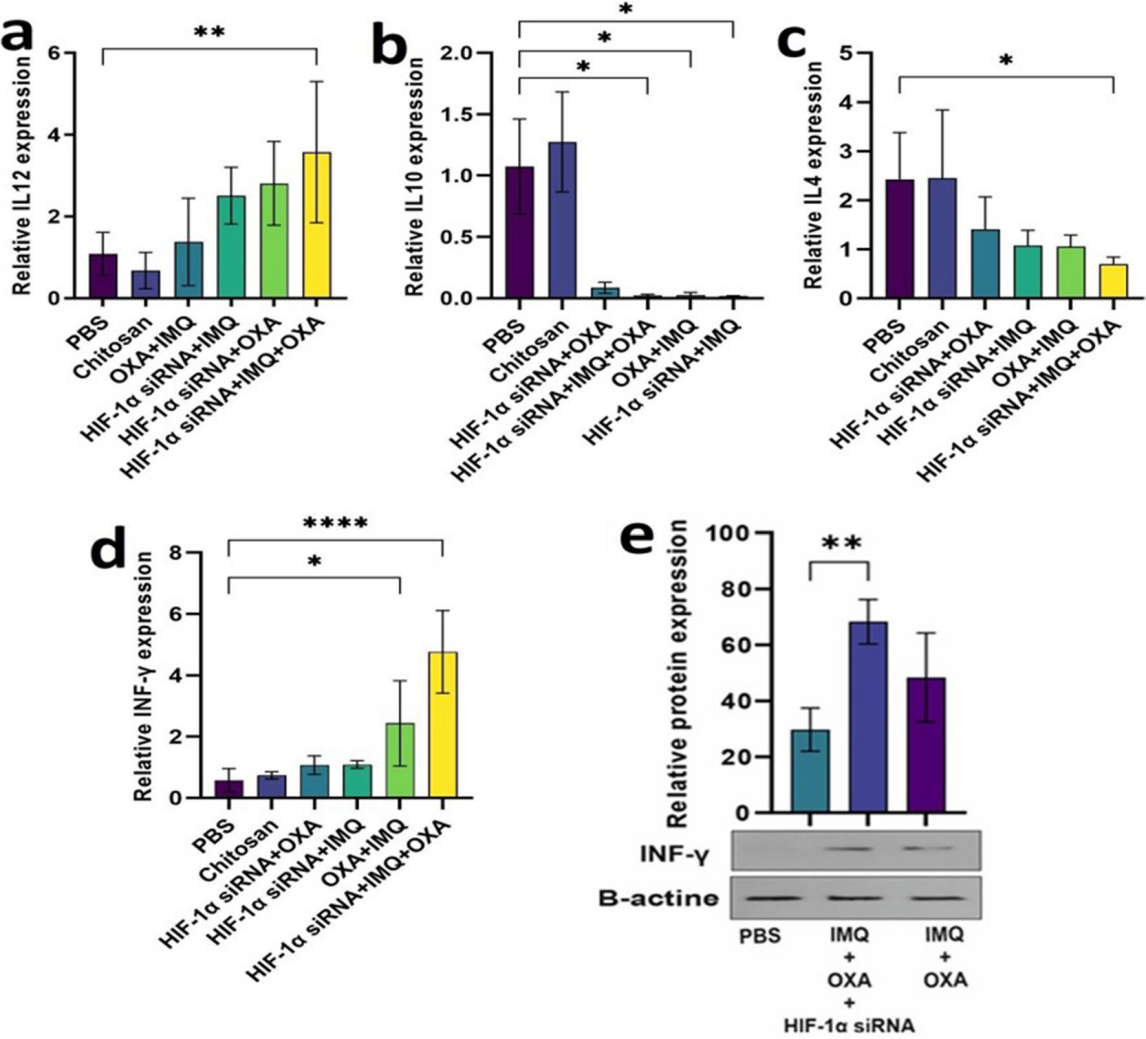
This figure investigates how combination therapy influences the expression of genes associated with cytokines. **a** to **c** show the mRNA and protein expression levels of interferon-gamma (IFN-γ). **d** and **e** show the mRNA expression levels of interleukin-12 (IL-12). Beta-actin, a housekeeping gene, was used for data normalization. All the data are presented as the mean ± standard deviation (SD) of the gene expression levels. Within the triple therapy group, HIF-1α expression showed a positive correlation with VEGF (r = 0.8), although this correlation was not statistically significant (Fig. 6b). In contrast, negative correlations were observed between HIF-1α expression and the expression levels of both BCL2 (Fig. 6c) and IL-12 (Fig. 6d). The strongest correlation (*p* = 0.09) was found between HIF-1α and BCL2 expression

To evaluate the effects of combination therapies on the immune system, the mRNA and protein levels of cytokines in treated and untreated tumor samples were analyzed. The mRNA expression levels of interferon-gamma (*IFN-γ*) were significantly higher in the triple treatment group than in the control group. A comparison between triple and double combination therapy revealed a significant increase in the *IFN-γ* gene in the triple combination therapy group when compared with all double treatment groups (Fig. 6a and b, *P*< 0.0001). Analysis of the protein expression levels revealed that the IFN-γ levels in the mice treated with triple combination therapy were higher than those in the control group. Additionally, the interleukin-12 (*IL-12*) mRNA expression levels were also significantly increased in the triple treatment group than in the control and IMQ + OXA groups (Fig. 6c-e , *P*= 0.01).

Compared with those in the control group, the expression levels of anti-inflammatory cytokines, such as interleukin-10 (*IL-10*), significantly decreased in all treatment groups except for the *HIF-1α* siRNA + OXA group. No significant differences in the mRNA expression levels of *IL-10* were observed between the triple- and dual-treatment groups. Moreover, the mRNA expression level of *IL-4 was* significantly lower in the tumor samples from the triple combination group than in those from the control and *HIF-1α* siRNA + OXA treatment groups (data not shown).

An overall comparison of all studied cytokine genes across treatment groups (Fig. 6j) revealed a predominant increase in genes associated with the cellular immune response (IFN-γ and IL-12) in the triple therapy group compared to the double therapy group. The expression of proapoptotic genes increased in all treatment groups, with the highest expression observed in the *HIF-1α* siRNA + OXA group. The expression levels of genes related to cell proliferation were considerably decreased in all tumor samples, with the lowest expression observed in the triple therapy group.

#### Correlation of HIF-1α gene expression with CT26 tumor growth phenotypes

**Fig. 7:**
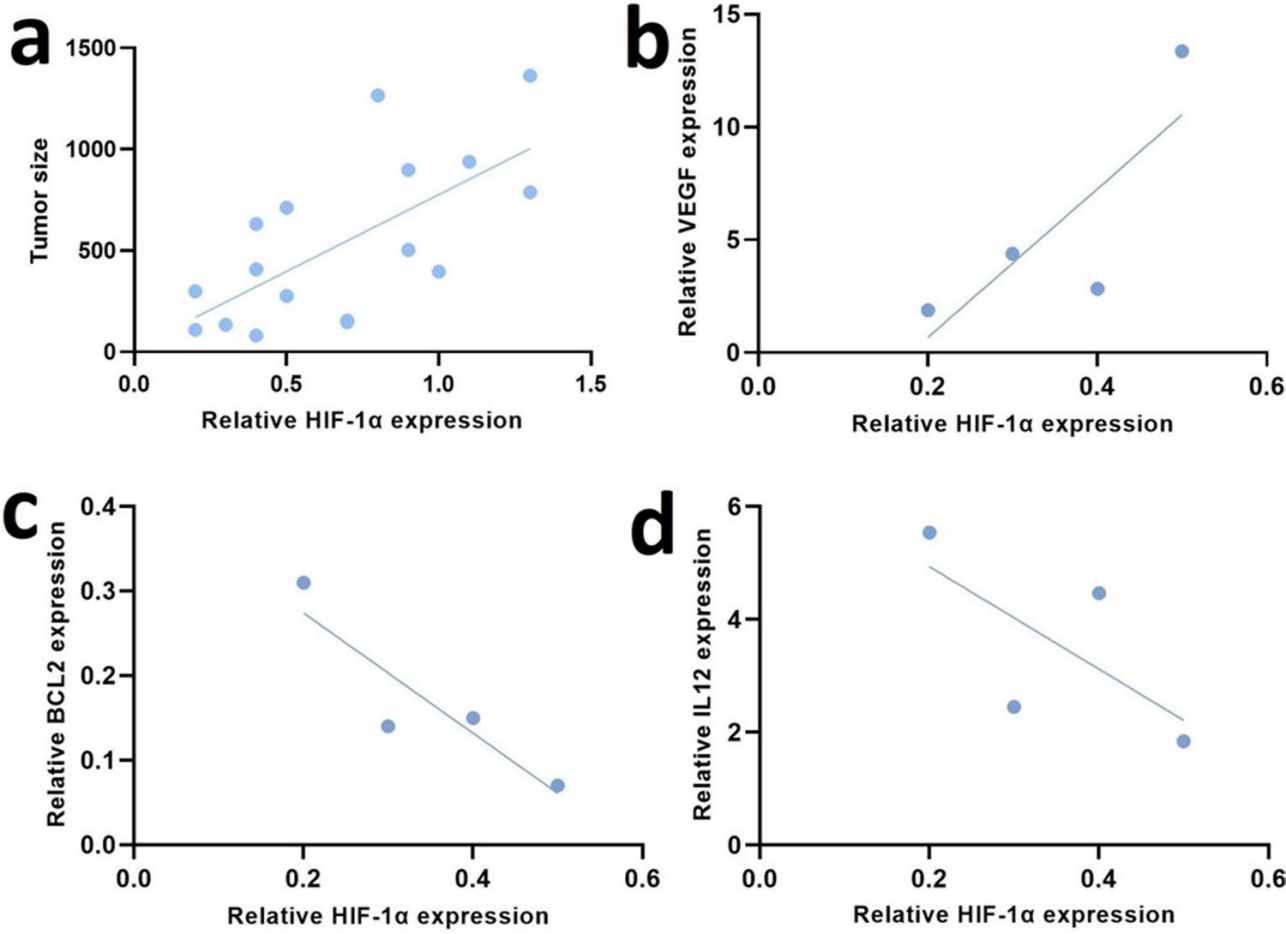
Correlation of HIF-1α Expression with Tumor Size and Other Genes. **a** The correlation between HIF-1α gene expression and tumor size across all treatment and control groups was explored. A positive correlation was observed, indicating that higher HIF-1α mRNA expression is associated with larger tumors. **b**-**d** The correlation between HIF-1α expression and the expression of other genes in the triple combination therapy group was investigated. **b** The correlations with vascular endothelial growth factor (VEGF), **c** BCL2, and **d** interleukin-12 (IL-12) were investigated

Spearman's correlation analysis was performed to determine the correlation between *HIF-1α* gene expression and tumor size in all tumor sample groups. As shown in Fig. 7a, the positive correlation coefficient (r = 0.68) between tumor size and *HIF-1α* expression levels was statistically significant (*P* = 0.003). In the triple combination therapy group, there was a positive correlation (r = 0.8) between *HIF-1α* and *VEGF*, although this correlation was not statistically significant (Fig. 7b, *p*=). Moreover, a negative correlation was observed between *BCL2*, *IL-12* and *HIF-1α* expression levels (Fig. 7c and d). The strongest genetic correlation (*P* = 0.09) was observed between *HIF-1α* and *BCL2*.

## Discussion

Our research revealed a new mechanism of tumor immune escape involving HIF-1α in combination with a Toll-like receptor 7 agonist and oxaliplatin. This study evaluated the expression of key genes and proteins associated with apoptosis, proliferation, and cellular immune responses in tumor-bearing mice treated with various combinations of HIF-1α siRNA, IMQ, and OXA. Several studies have demonstrated that drug delivery-based nanoparticles are ideal candidates for siRNA delivery in vivo (Yu et al. 2017). Chen et al. showed that combining hypoxia-inducible factor (HIF)−1α siRNA delivered via lipid-calcium phosphate nanoparticles (LCPs) with photosan-mediated photodynamic therapy (PDT) effectively suppressed the growth of head and neck tumors both in vitro and in vivo (Chen et al. 2015). It seems that utilizing these chitosan polymers could also improve cellular absorption by enhancing the condensation of nucleic acids into nanoparticles. Our results indicated that *HIF-1α* siRNA could be effectively encapsulated in CH nanoparticles. In addition, the decrease in zeta potential to 12.2 ± 0.4 mV after siRNA incorporation confirmed the successful formation of the CH/siRNA nanoplexes. The uniformity of size and shape of nanoparticles were confirmed by nanoparticles sizer and SEM micrographs, respectively. Furthermore, no impurities were observed in the SEM analysis. Various experimental and clinical studies have reported that elevated levels of *HIF-1α* are associated with an immunosuppressive TME, rendering it resistant to therapy and cancer progression (Schmitz et al. 2009, Sowa et al. 2017, Xu et al. 2019). Izadi et al. showed that simultaneous delivery of HIF-1α siRNA and Dinaciclib using graphene oxide-trimethyl chitosan-hyaluronate effectively inhibited CT26 colorectal cancer and B16-F10 melanoma cancer cell progression (Izadi et al. 2020). Therefore, targeting *HIF-1α* along with other therapies has recently emerged as an attractive strategy to increase therapeutic sensitivity and suppress the aggressive phenotype of tumors (Imamura et al. 2009, Li et al. 2015). Lequeux et al. demonstrated that targeting the *HIF-1α* pathway can switch the immunosuppressive TME to an immune-supportive TME through the activation of the cellular immune system, leading to tumor growth inhibition (Lequeux et al. 2021). Our results showed that i.t. injection of *HIF-1α* siRNA in combination with IMQ and OXA successfully induced tumor cell apoptosis and inhibited the growth of a syngeneic mouse model of CRC. Our results indicated increased efficiency of IMQ and OXA in mouse models of CRC when *HIF-1α* was downregulated by siRNA, which is in agreement with the findings of Ying Li et al., showed that inhibiting the growth of SK-NEP-1 Wilms tumor cells in vitro and suppressing tumorigenesis and angiogenesis in vivo can be achieved by silencing hypoxia-inducible factor-1α (Shi et al. 2016). Compared to chemoimmunotherapy (IMQ + OXA), which led to the largest increase in tumor size, the incorporation of HIF-1α siRNA resulted in the smallest increase in tumor size. Among the treatment groups, triple combination therapy was found to have the highest antitumor efficacy and a strong inhibitory effect on tumor growth in a syngeneic mouse model of CRC. It is postulated that triple combination therapy likely inhibits tumor growth through several mechanisms, including potent immune responses against tumor cells, inhibition of cell proliferation, and induction of apoptosis.

In our study, we found that the co-administration of OXA and *HIF-1α* siRNA inhibited cell proliferation, induced tumor cell apoptosis, and slowed tumor growth, consistent with previous studies (Li et al. 2015, Ai et al. 2016). Our findings showed that targeting *HIF-1α* with OXA inhibited tumor cell growth via direct cytotoxicity rather than immunoregulation. Therefore, these data suggest that the presence of a favorable TME or a sufficient amount of TAA alone may not be sufficient to stimulate a potent antitumor immune response and modify the suppressed immune system. Combining these agents with an immune stimulant may induce a further antitumor immune response.

Immunotherapy with TLR agonists, alone or in combination with conventional treatments, has been widely studied for cancer therapy. Multiple studies have demonstrated that targeting TLR7 leads to strong recruitment and activation of immune cells in the TME and shifts the Th1 immune response, which inhibits tumor growth in various syngeneic mouse models (Mullins et al. 2019). The FDA-approved agent IMQ, a TLR7 agonist, is currently used for the treatment of dermatological cancers, such as basal cell carcinoma (BCC), and viral lesions, such as human papillomavirus (HPV). It seems that the activation of immune cells is influenced by HIF overexpression, which is associated with immunosuppressive TME markers. The combination of an *HIF-1α* inhibitor and ICIs promotes tumor cell apoptosis and antitumor efficacy in cancer immunotherapy, leading to enhanced tumor growth inhibition in lung (Luo et al. 2022) and prostate cancer mouse models (Shen et al. 2022). Our results showed that mice treated with IMQ in combination with *HIF-1α* siRNA exhibited a significant increase in the expression of apoptotic genes and a decrease in the expression of the proliferative genes *VEGF* and *p-STAT3*, leading to the inhibition of tumor growth. In addition, compared with triple therapy, *HIF-1α* siRNA improved the efficacy of IMQ in activating the immune system against tumor growth; however, this effect was associated with fewer immunomodulatory effects.

Several studies have reported synergistic therapeutic effects of combining chemotherapy with multiple TLR agonists (Rostamizadeh et al. 2022). Direct intratumoral administration allows for the maximization of the therapeutic index of various immunomodulatory therapies by minimizing systemic exposure. Essentially, this approach aims to stimulate the immune system directly at the tumor site, effectively using the tumor as its own vaccine to initiate or boost an existing antitumor immune response. Previous preclinical and clinical studies suggest that PRR agonists and oncolytic viruses show increased activity when delivered locally and may work synergistically with systemic immune-checkpoint inhibitors (Aznar et al. 2017). Chemotherapy drugs generate an environment saturated in tumor-associated antigens (TAAs) that can be triggered by Toll-like receptors (TLRs), thus promoting the activation of immune cells and fostering an antitumor immune reaction (Lamberti et al. 2020). Molecular analysis revealed that the expression levels of cytokines related to the cellular immune response (*IL12* and *IFN-γ*) were significantly lower in the IMQ and OXA treatment groups than in the triple combination therapy group. These results highlight the roles of HIF inhibition in enhancing therapeutic responses.

Compared with dual combination therapy, triple combination treatment strongly upregulated Th1 (T helper 1)-type cytokines, including *IL-12* and *IFN-γ*, but not Th2-type cytokines, such as *IL-4* and *IL-10*. These results are consistent with the idea that the co-administration of *HIF-1α* siRNA + OXA + IMQ elicited cellular immune responses in tumor-bearing mice. This effect is likely due to the improved function of immune cells stimulated by IMQ and OXA in the favorable microenvironment induced by *HIF-1α* siRNA. Our results showed that despite the decreased proliferation and angiogenesis genes in all the combined treatments, the lowest tumor size was observed in the triple treatment group. This suggests that the inhibition of *HIF-1α* gene expression and the establishment of a favorable TME are essential for optimal immune cell function.

## Conclusion

Taken together, our findings highlight the potential beneficial effects of triple combination therapy in a mouse model of CRC. Combination therapy capable of diminishing *HIF-1α* levels and boosting the immune response can be considered a promising therapeutic strategy for colon cancer treatment.

## Supplementary Information

Below is the link to the electronic supplementary material.

Supplementary file1 (DOCX 357 KB)

## Data Availability

All source data for this work (or generated in this study) are available upon reasonable request.
