# Supplementary figures and images for: Modulating the tumor microenvironment in a mouse model of colon cancer using a combination of HIF-1α inhibitors and Toll-Like Receptor 7 agonists
Source: Naunyn Schmiedebergs Arch Pharmacol. 2024 Nov 30;398(5):5867–80. doi: 10.1007/s00210-024-03658-8 (PMC11985627; doi:10.1007/s00210-024-03658-8)

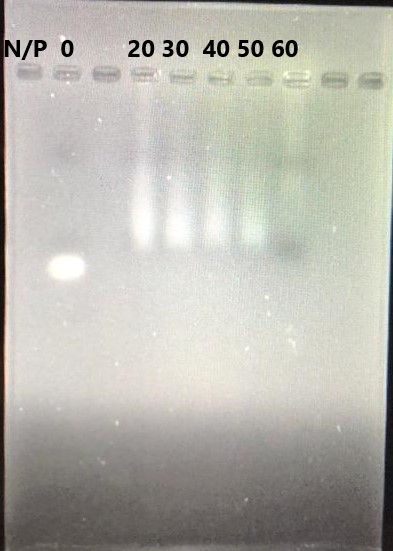


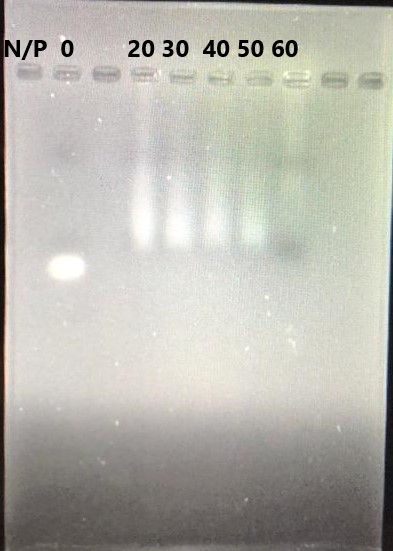


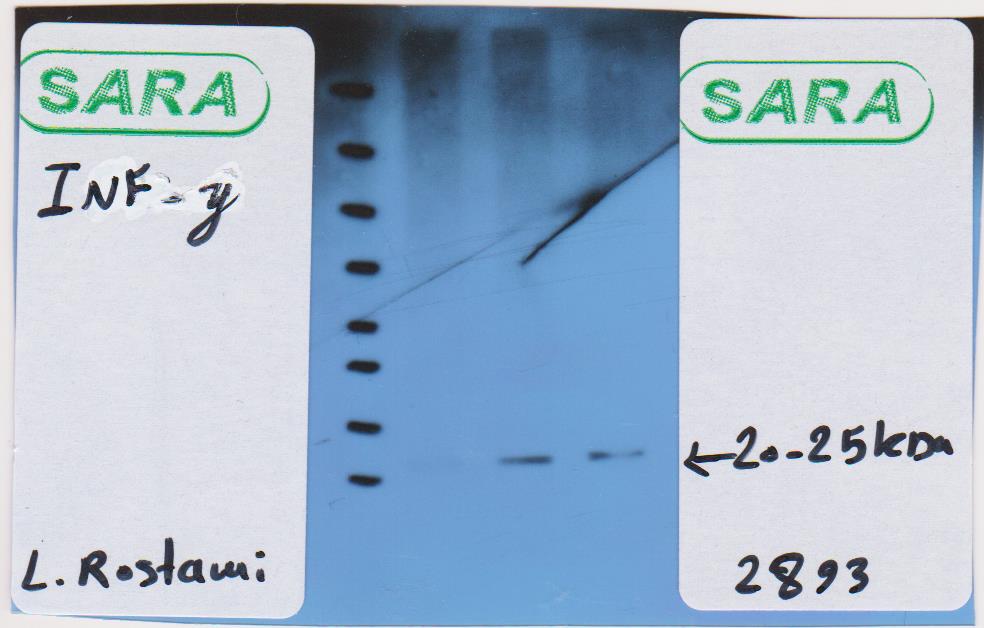

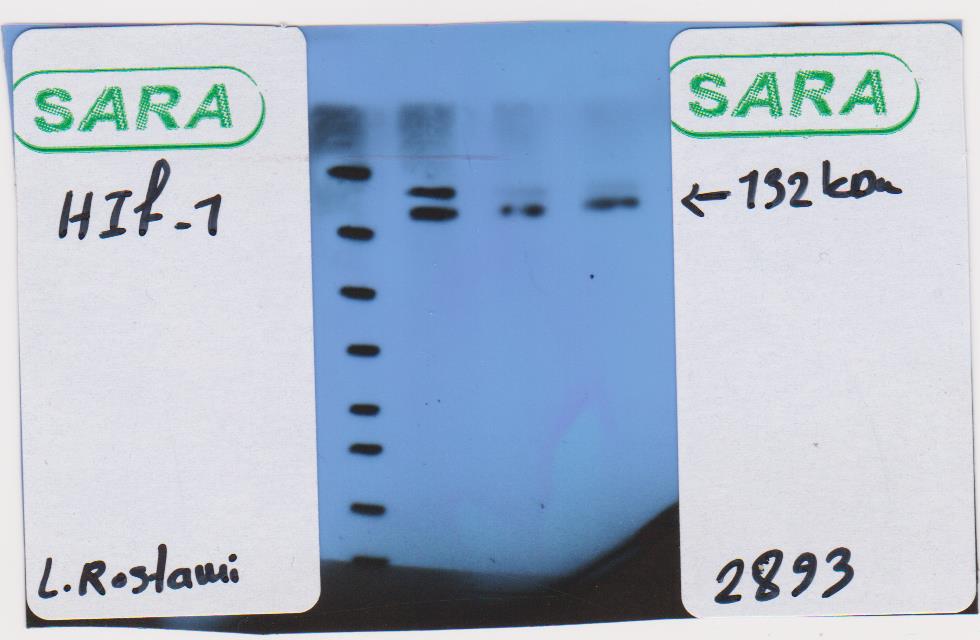

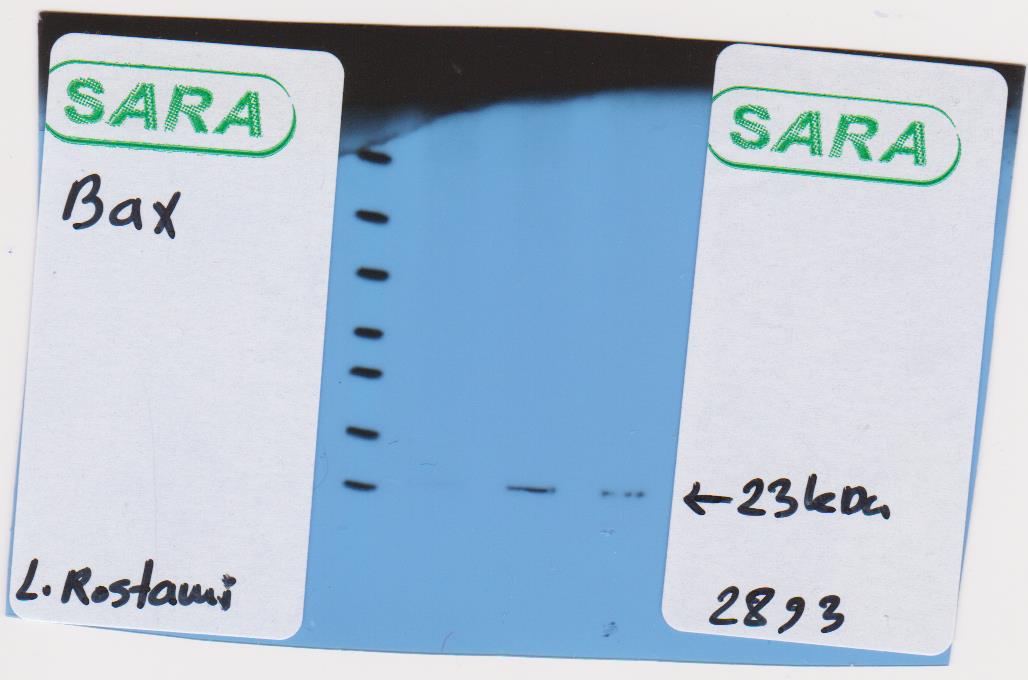

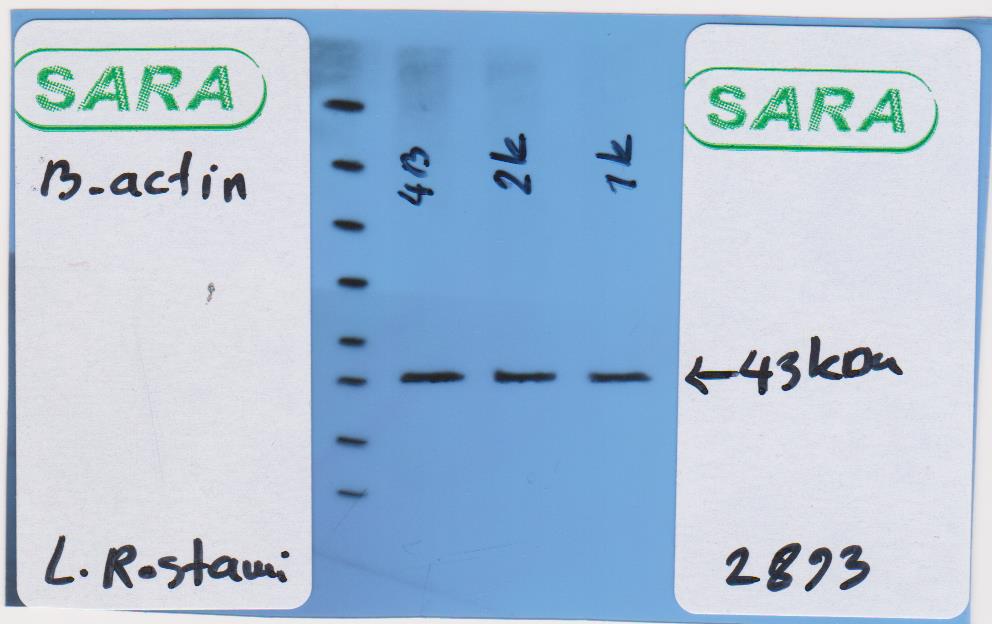

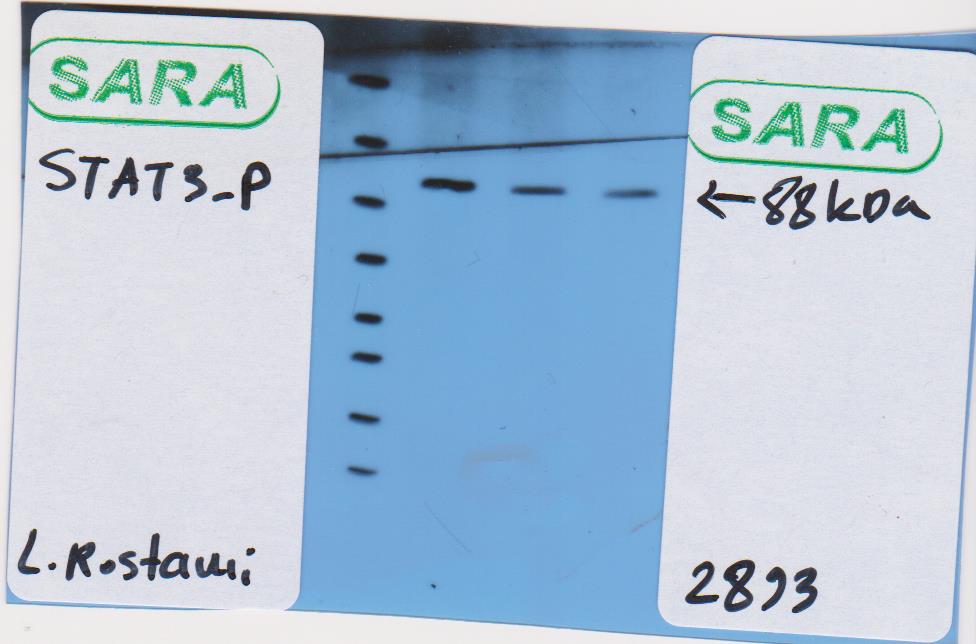

Supplement: Supplementary file 1 — Supplementary file1 (DOCX 357 KB) [file 210_2024_3658_MOESM1_ESM.docx]
